# Ultrathin Carbon-Coated Porous TiNb_2_O_7_ Nanosheets as Anode Materials for Enhanced Lithium Storage

**DOI:** 10.3390/nano12172943

**Published:** 2022-08-26

**Authors:** Dewei Liang, Yu Lu, Ningning Zhou, Zezhong Xu

**Affiliations:** School of Energy Materials and Chemical Engineering, Hefei University, Hefei 230601, China

**Keywords:** carbon-coated, TiNb_2_O_7_ nanosheets, porous nanostructure, synergistic effect, anode material

## Abstract

TiNb_2_O_7_ has been considered as a promising anode material for next-generation high power lithium ion batteries for its relatively high theoretical capacity, excellent safety and long cycle life. However, the unsatisfactory electrochemical kinetics resulting from the intrinsic sluggish electron transport and lithium ion diffusion of TiNb_2_O_7_ limit its wide application. Morphology controlling and carbon coating are two effective methods for improving the electrochemical performance of electrode materials. Herein, an ultrathin carbon-coated porous TiNb_2_O_7_ nanosheet (TNO@C) is successfully fabricated by a simple and effective approach. The distinctive sheet-like porous structure can shorten the transport path of ions/electrons and provide more active sites for electrochemical reaction. The introduction of nanolayer carbon can improve electronic conductivity and increase the specific surface area of the porous TiNb_2_O_7_ nanosheets. Based on the above synergistic effect, TiNb_2_O_7_@C delivers an initial discharge capacity of 250.6 mAh g^−1^ under current density of 5C and can be maintained at 206.9 mAh g^−1^ after 1000 cycles with a capacity retention of 82.6%, both of which are superior to that of pure TiNb_2_O_7_. These results well demonstrate that TiNb_2_O_7_@C is a promising anode material for lithium ion batteries.

## 1. Introduction

Given the merits of high energy density and good cycle life, lithium-ion batteries (LIBs) have been rapidly used in portable electronic equipment and electric vehicles [1,2,3]. Graphite has been marketed as a commercial anode material for LIBs; however, its low operating potential plateau (~0.2 V vs. Li^+^/Li) easily leads to the formation of solid electrolyte interphase (SEI) and lithium dendrites in the process of charging and discharging, and further leads to irreversible capacity degradation and security risks [4,5]. Therefore, the development of new materials with higher capacity, more excellent rate and cycle performance, as well as higher safety, has attracted more and more attention.

Intercalation type anode material Li_4_Ti_5_O_12_ has been widely studied and already used in commercial fast-charging LIBs owing to its high voltage discharge plateau (~1.55 V vs. Li/Li^+^) and “zero-strain” characteristic, which can avoid the formation of the SEI layer and lithium dendrites during rapid charge/discharge [6,7]; however, the low theoretical capacity (175 mAh g^−1^) of Li_4_Ti_5_O_12_ restricts its further application. Recently, TiNb_2_O_7_ (denoted as TNO) has been considered as a promising anode material to replace Li_4_Ti_5_O_12_ due to its relatively high theoretical capacity (387 mAh g^−1^) and higher voltage plateau (~1.65 V vs. Li/Li^+^), thus improved security and higher capacity are both achieved [8,9]. The TNO with ReO_3_ structure consists of 3 × 3 × ∞ blocks, where Ti^4+^ and Nb^5+^ ions are randomly spread in the octahedral sites linked by edges and corners. The unique open-tunneling ion channel facilitates high-rate diffusion of lithium ions within the crystal structure [9]. Unfortunately, the inherent low electronic conductivity (<10^−9^ S cm^−1^) [10] and sluggish Li^+^ diffusivity (<10^−17^ cm^2^ s^−1^) [11,12] of TNO makes its electrochemical performance unsatisfactory, hindering its large-scale practical application in LIBs.

To boost the electrochemical performance of TNO, researchers have adopted a variety of methods [13,14,15,16,17,18]. The first strategy is to construct a nanoscale structure to shorten the transmission path of Li^+^, especially for building two-dimensional porous nanostructures [19,20,21,22,23,24], which can further offer higher specific surface area, more active sites, as well as relieve volume change, thus realizing superior electrochemical performance. Carbon coating is another effective method for modifying the electrochemical properties of electrode materials [12,25,26]. However, excessive carbon content will inevitably reduce the volume specific capacity of the material, hence appropriate thickness of the carbon layer is more suitable for the comprehensive improvement of the electrochemical performance of electrode materials. With the development of nanomaterial modification strategies, researchers usually use two modification strategies together to modify the target material, and the synergistic effect of the two modification methods is more beneficial for enhancing its electrochemical performance [27,28,29]. However, their synthesis methods are mostly time consuming, high cost or require template assistance, increasing the cost of practical application. In view of all this, developing a simple and low-cost method to achieve ultrathin carbon coating is important for the modification of nanomaterials.

Herein, we first report a nanolayer carbon-coated porous TNO nanosheet fabricated using a simple and effective in situ surface adsorption and carbonization approach with furfuryl alcohol as the carbon precursor. As an anode in LIBs, TNO@C electrodes exhibit enhanced electrochemical performance compared to unmodified TNO, including delivering a high capacity of 250.6 mAh g^−1^ at 5C, and can be maintained at 206.9 mAh g^−1^ after 1000 cycles with a capacity retention of 82.6%, both of which are superior to pure TNO, revealing that TNO@C can be a promising anode with excellent safety for high-rate LIBs.

## 2. Experimental

### 2.1. Preparation of TNO@C Nanosheets

Porous TNO nanosheets were prepared according to our previous research [30]. A total of 0.4 g of TNO powder was added into a mixture of 5% v/v furfuryl alcohol and alcohol, and sufficient ultrasonic and continuous magnetic stirring was carried out to ensure the full adsorption of furfuryl alcohol onto the surface of TNO nanosheets. The product was then centrifuged from the solution and dried at 80 °C for 6 h. Subsequently, the black powder was obtained after heating the samples at 600 °C for 2 h in Ar atmosphere and named as TNO@C.

### 2.2. Characterization of Materials

X-ray powder diffractometry (XRD, Smart Lab, Rigaku Co., Tokyo, Japan) with a wavelength of 1.5406 Å was employed to analyze the phase of the samples. The scanning electron microscopy (SEM, Hitachi Co., Tokyo, Japan) images were taken with a SU8010 field emission scanning electron microscope. Transmission electron microscopy (TEM, JEOL Ltd., Tokyo, Japan) images were taken with a JEOL-2010 transmission electron microscope. The nitrogen adsorption and desorption isotherms and pore size distributions were characterized by a Micromeritics ASAP 2020 plus analyzer (Micromeritics Instrument Co., GA, USA). The thermalgravimetric analysis (TGA, Q500, TA Instrument Co., Delaware, USA) was performed in flowing air from 30 °C to 800 °C to study the carbon content in composite. The structural characteristics of the carbonaceous materials in the product were investigated by Raman spectroscopy (LabRAM HR Evolution, HORIBA Co., Kyoto, Japan) excited by a 532 nm laser.

### 2.3. Electrochemical Measurements

The working electrode was prepared by grinding the active material, Super P and polyvinylidene fluoride at a weight ratio of 7:2:1, then a certain amount of 1-methyl-2-pyrrolidone (NMP) was added, followed by ball-milling using a planetary ball-milling apparatus. The slurry was then pasted onto the copper foil with a coating thickness of 100 μm. The obtained film was dried under vacuum at 120 °C for 5 h, then cut into thin discs with a of diameter 12 mm. The area load of the TiNb_2_O_7_@C for the obtained electrode was ~1.0 mg cm^−^^2^. CR2016 coin-type cells were assembled in an Ar-filled glove box. Lithium foil and polypropylene membrane (2400, Celgard) were employed as the counter electrode and separator, respectively. A total of 1 mol L^−1^ LiPF_6_ in an ethylene carbonate (EC) and dimethyl carbonate (DMC) mixture (1:1 *v*/*v*) was utilized as the electrolyte. Cyclic voltammetry (CV) and electrochemical impedance spectroscopy (EIS) were executed on a two-electrode system of a Zahner electrochemical workstation (Zahner Elektrik, Kronach, Germany), and the frequency range of EIS tests was from 100 kHz to 0.1 Hz. Cycling performance was measured at room temperature on a Land CT2001A instrument (Land, Wuhan, China). Specific capacity was measured and calculated based on the actual mass of the active material, excluding additional carbon and binder weight.

## 3. Results and Discussion

**Figure 1:**
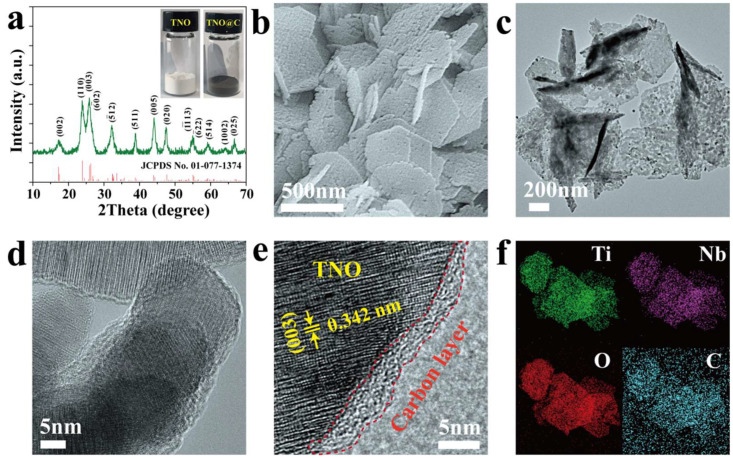
(**a**) XRD pattern of the TNO@C sample. The inset shows optical images of the TNO and TNO@C powders; (**b**) SEM image of TNO@C; (**c**) TEM image of TNO@C; (**d**–**f**) HRTEM images and EDX mapping of TNO@C.

Figure 1a plots the XRD patterns of the as-synthesized sample, which indicates the formation of monoclinic phase TiNb_2_O_7_ (JCPDS No. 01-77-1374). No other impurity peaks could be observed, suggesting the as-prepared sample was phase-pure TNO, which was consistent with previous research [30]. The absence of the characteristic peak of carbon in TNO@C might be due to the amorphous carbon or low amount of carbon. The inset of Figure 1a shows the optical images of the TNO and TNO@C powders and, as can be seen from the picture, the color of the TNO changed from white to black after the carbon coating treatment, which further confirms the surface of TNO had been successfully coated with carbon.

Figure 1b shows the surface morphology of TNO@C, which also showed a sheet-like nanostructure and similar size, length and width compared to the pure TNO in our previous study [30], indicating the surface carbon coating process had no effect on the structure of the TNO material. Figure 1c presents the TEM image of the TNO@C that further confirms the existence of a porous structure, which could shorten the migration path for ions during the charge and discharge process. Figure 1d and 1e show the HRTEM image of the TNO@C, and ultrathin graphitized carbon layers can be seen appearing around the edge of nanosheets. The lattices, with spacing of 0.342 nm, corresponded to the (003) crystal planes of monoclinic phase TiNb_2_O_7_ and, when combined with the uniform distribution of the Nb, Ti, O and C elements throughout the sample area in the EDX mappings (Figure 1f), further confirms the successful coating of ultrathin carbon on the surface of TNO nanosheets.

**Figure 2:**
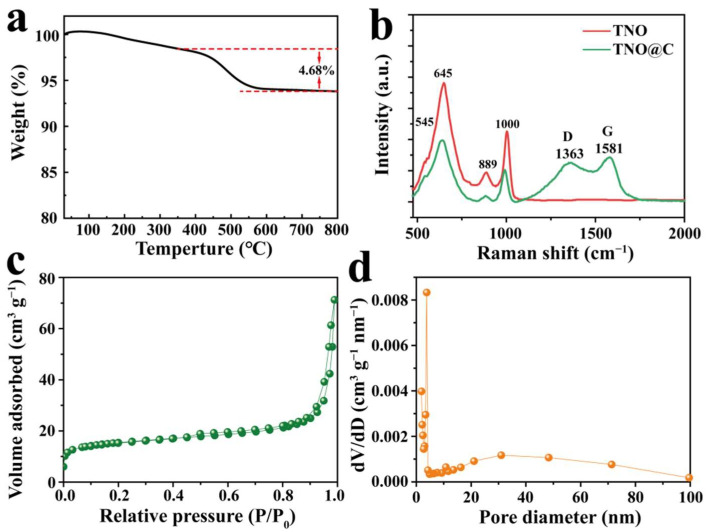
(**a**) TG curve of TNO@C analysis performed in air; (**b**) Raman spectra of TNO and TNO@C; (**c**,**d**) N_2_ adsorption–desorption isotherm and pore-size-distribution curve of the TNO@C.

The thermogravimetric analysis was carried out in air to study the carbon content in the composite. As shown in Figure 2a, the carbon content in the TNO@C was calculated to be 4.68%, and the weight loss occurring from 350 °C to 800 °C corresponds to the oxidation of carbon [8]. Figure 2b showed the Raman spectra of the TNO and TNO@C. The peaks at 545 and 645 cm^−1^ can be attributed to the occupied edge-shared TiO_6_ octahedra and the peaks at 889 and 1000 cm^−1^ can be attributed to the corner/edge-shared NbO_6_ octahedra, respectively [25]. For the TNO@C sample, another two peaks at 1363 and 1581 cm^−1^ can be attributed to graphitic carbon’s D and G bands, respectively. The intensity ratio of these peaks (I_D_/I_G_) was ~0.88, showing that the degree of graphitization for the coated carbon was relatively high [3]. In addition, the characteristic peak intensity of the TNO@C sample became lower compared to a pure TNO nanosheet, also demonstrating the successful coating of carbon [17]. Figure 2c shows the specific surface area of TNO@C was 51.60 m^2^ g^−1^, which was higher than that of TNO (32.08 m^2^ g^−1^) in our previous study [30]. Figure 2d shows the average BJH pore diameter distribution curve of TNO@C based on desorption curves. It can be seen from the picture that the TNO@C showed a rich mesopore with pore-sizes centered at 3.8 nm, mainly derived from greatly reduced inter-particle pores after the uniform surface coating of carbon layer.

**Figure 3:**
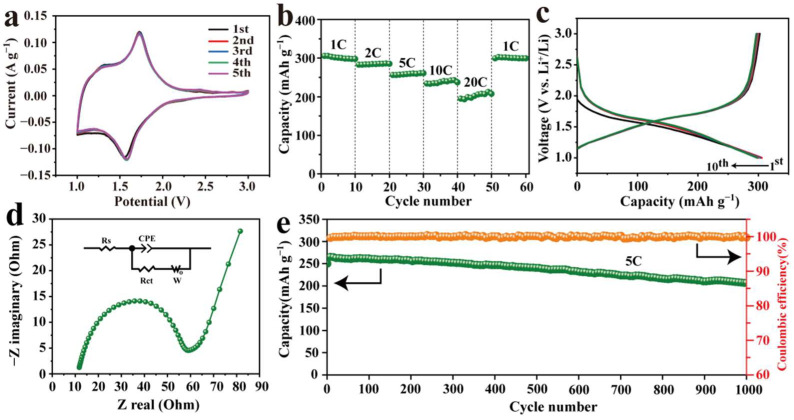
(**a**) Initial five CV curves of TNO@C at a 0.1 mV s^−1^ scan rate; (**b**) the rate performance of TNO@C; (**c**) constant current discharge/charge curve of the ten initial cycles of TNO@C at 1 C; (**d**) Nyquist plot of the TNO@C electrode; (**e**) plot of discharge capacity versus cycle number.

Figure 3a shows the CV curves of TNO@C electrodes at a scan rate of 0.1 mV s^−1^. Note that TNO@C exhibited a sharper pair of redox peaks at 1.57 and 1.72 V, which corresponds to the reversible conversion of Nb^5+^/Nb^4+^. Moreover, an extremely faint pair of shoulder peaks from 1.0 to 1.4 and from 1.7 to 1.9 V could refer to the Nb^4+^/Nb^3+^ and the Ti^4+^/Ti^3+^ redox couples [31], respectively. The five cyclic voltammogram curves of TNO@C showed high comparability, suggesting minor irreversible capacity loss and small polarization. The lithium insertion/extraction reaction mechanism was represented as [32]:

(1) $$\begin{array}{l} TiNb_{2}O_{7}+xLi^{+}+xe^{-}↔Li_{x}TiNb_{2}O_{7}\\ 0\leq x\leq 5 \end{array}$$

Figure 3b presents the rate performance of the TNO@C electrode. As current density increased from 1 to 2, 5, 10 and then 20 C before returning to 1 C, the corresponding average reversible discharge capacities were 301.0, 284.4, 258.7, 237.7, 202.5 and 300.6 mAh g^−1^ for TNO@C, respectively. The capacity and stability of the TNO@C electrode is much higher than that of the pure TNO electrode at high current density from our previous study [30], demonstrating the nanolayer carbon coating could effectively improve the rate performance and stability of TNO. Figure 3c shows galvanic discharge/charge curves of the first ten cycles of the TNO@C at 1 C. Two slope regions located at 3.0–1.7 and 1.5–1.0 V were indicative of a solid solution reaction, with a discharge plateau of approximately 1.65 V [33]. The first coulombic efficiency of TNO@C was 98.2%, which is much higher than that of the TNO (75.7%) used in our previous research [30].

The electrochemical impedance spectra (EIS) of TNO@C were measured and are shown in Figure 3d. The Nyquist plot shows a semicircle and sloping straight line in the high frequency and low frequency region, respectively. The semicircle represents charge transfer resistance (R_ct_) and the sloping straight line reflects the diffusive power of Li^+^ [34]. TNO@C showed a charge transfer resistance of 59 Ω, which is lower than that of the TNO (220 Ω) used in our previous research [30], reflecting the significant effect of carbon coating on reducing the charge transfer resistance of electrodes. However, the contact resistance value (12 Ω) of TNO@C was higher, which might be due to agglomeration between the nanosheets after carbon coating modification. Figure 3e displays the cycling performance of TNO@C at 5 C (~1.94 A g^−1^) current density. The initial capacity of TNO@C was 250.6 mAh g^−1^. After 1000 cycles, the capacity remained 206.9 mAh g^−1^ with a capacity retention of 82.6%, showing better reversibility and stability regarding cycling performance than that of the pure TNO (initial capacity of 245.8, and 70.2% capacity retention after 1000 cycles) used in our previous research [30], owing to the coated nanolayer carbon providing a conductive network for the fast transfer of electrons. Structural defects, crystallinity, test parameters (current density, voltage range), formed SEI film or other factors might retard Li^+^ diffusion and further lead to the measured capacity of TNO and TNO@C being lower than that of the theoretical capacity of TNO [35].

**Figure 4:**
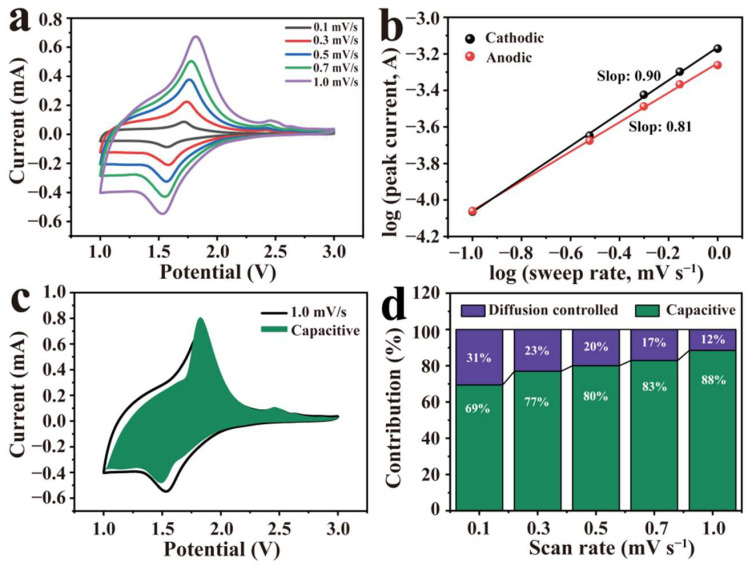
(**a**) CV curves and (**b**) b values of peak currents of the TNO@C at different scan rates; (**c**) capacitive contribution of TNO@C at 1.0 mV s^−1^; (**d**) capacitive contribution percentage of TNO@C.

To further explore the electrochemical kinetics of the TNO@C electrode, CV curves were recorded with increased scan rates as shown in Figure 4a. The peak current (*i*) and the scan rate (*v*) had the following relationship [36]:

(2) $$i=\alpha \nu^{b}$$

where *a* and *b* were adjustable parameters and the value of b could be deducted from the slope of the log(*i*) vs. log(*v*) curve. If *b* approached 0.5, indicating a diffusion-controlled electrochemical process. When *b* was equal to 1.0, it represented a capacitive-controlled reaction [37]. Fitting results showed the calculated b values were 0.90 (cathodic) and 0.81 (anodic) for the TNO@C electrode (Figure 4b), which reveals that both lithium ion intercalation and surface capacitance existed in its electrochemical reaction, and the proportion of the capacitive-controlled reaction of TNO@C is higher than that of TNO (the fitted b values of the cathodic and anodic peak currents in our previous study [30] were 0.68 and 0.71, respectively), owing to the increased specific surface area after the carbon coating modification.

To quantitatively determine the capacitive contribution of TNO@C to electrochemical performance at a certain scanning rate, Equation (2) was converted to:

(3) $$i=k_{1}v+k_{2}v^{1/2}$$

where *k*_1_ and *k*_2_ are adjustable values, *k*_1_*v* is the surface-controlled capacitive behavior and *k*_2_*v*^1/2^ is diffusion-controlled processes [25]. The green area indicating surface capacitive contribution was present at 1.0 mV s^−1^ in the CV curve (Figure 4c). Furthermore, a schematic diagram displayed the percentages of the capacitive contributions of the TNO@C electrode at different scanning rates, as exhibited in Figure 4d, and showed 69, 77, 80, 83 and 88% capacitive contribution at scan rates of 0.1, 0.3, 0.5, 0.7 and 1.0 mV s^−1^, respectively, which was helpful for enhancing the rate performance of the TNO@C electrode.

## 4. Conclusions

To sum up, a simple and effective approach was proposed to prepare ultrathin carbon-coated porous TNO nanosheets using furfuryl alcohol as a carbon precursor. Owing to the synergistic effect of the nanolayer carbon coating and the novel porous nanosheet structure, the TNO@C possessed specific capacities of 258.7, 237.7 and 202.5 mAh g^−1^ at 5, 10 and 20 C, respectively, and exhibited excellent capacity retention of 82.6% after 1000 cycles at 5 C, both of which are more excellent than that of pure TNO. Thus, TNO@C could be a promising anode material for high-security and high-rate lithium ion batteries, and the effective carbon coating route could also be extended to other materials.

## Data Availability

Not applicable.
